# 1207. Coronavirus Disease, 2019 (COVID-19) in Long-Term Care Facilities (LTCF): One Large County’s Response, California 2020-2021

**DOI:** 10.1093/ofid/ofab466.1399

**Published:** 2021-12-04

**Authors:** Raymond Y Chinn, Sayone Thihalolipavan, Jennifer Wheeler, Grace Kang, John D Malone, Eric McDonald

**Affiliations:** 1 County of San Diego, Health and Human Services Agency, San Diego, California; 2 County of San Diego Health and Services Agency, San Diego, California; 3 County of San Diego, San Diego, California; 4 County of San Diego, Epidemiology & Immunization Services Branch, San Diego, California

## Abstract

**Background:**

The coronavirus-19 disease (COVID-19) outbreak has had a particularly devasting effect on skilled nursing facility (SNF) residents and healthcare workers (HCWs). While representing only 11% of COVID-19 cases, the residents accounted for 43% of deaths in the United States.

**Methods:**

We report a retrospective review of the support provided by our local health department (LHD) to long-term care facilities in response to the COVID-19 pandemic. This group comprised of staff from healthcare-associated infections (HAI); the Medical Operations Center (MOC); Testing, Tracing, and Treatment (T3); and the Healthcare Provider Status Taskforce (Table 1 outlines their functions). The HAI team with the State Public Health Department provided infection prevention and control (IPC) outbreak investigation, education, recommendations, and ongoing access to technical assistance. The T3 team focused on rapid response testing and tracing; the HPSTF team collected data and issued questionnaires; the MOC responded to staffing and PPE requests; and the Long-Term Care Facility sector presented routine telebriefings to update the facilities on public health guidance, share resources, and answer questions during and in between briefings.

Table 1. Sectors and Function of Response Teams to COVID-19

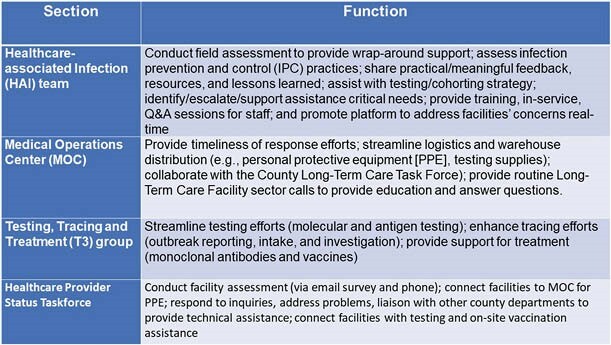

**Results:**

From March 2020 through May 2021, there were 504 outbreaks in LTCFs; the HAI team performed 281 outbreak investigations (Figure 1). In the same period, 308,264 molecular tests were performed using various platforms; laboratory services were outsourced during peak testing requests (Figure 2); “strike teams were deployed to facilitate testing on 404 occasions. Self-reported fully vaccination rate for SNF staff was 73% (March 2021) and 76% for residents (April 2021). There were 568 staff requested; total orders for PPE were 4,839 and 16,892,823 PPE items were fulfilled (Figure 3). In addition to knowledge gaps in IPC, other challenges included shifting IPC guidance, PPE shortages, timeliness of test results that impacted cohorting, community acquisition of disease with transmission to residents, interfacility spread among staff, staffing shortages, and vaccine hesitancy issues.

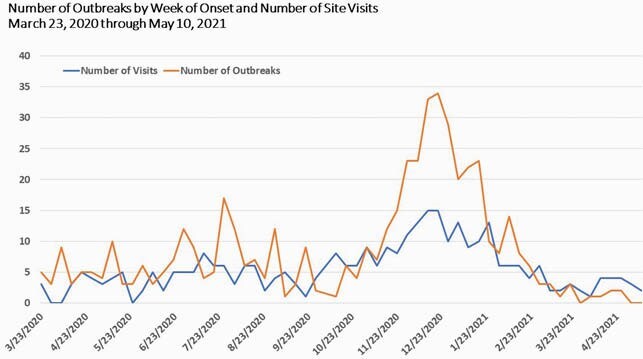

Figure 1. Number of Outbreaks and Number of Outbreak Investigations

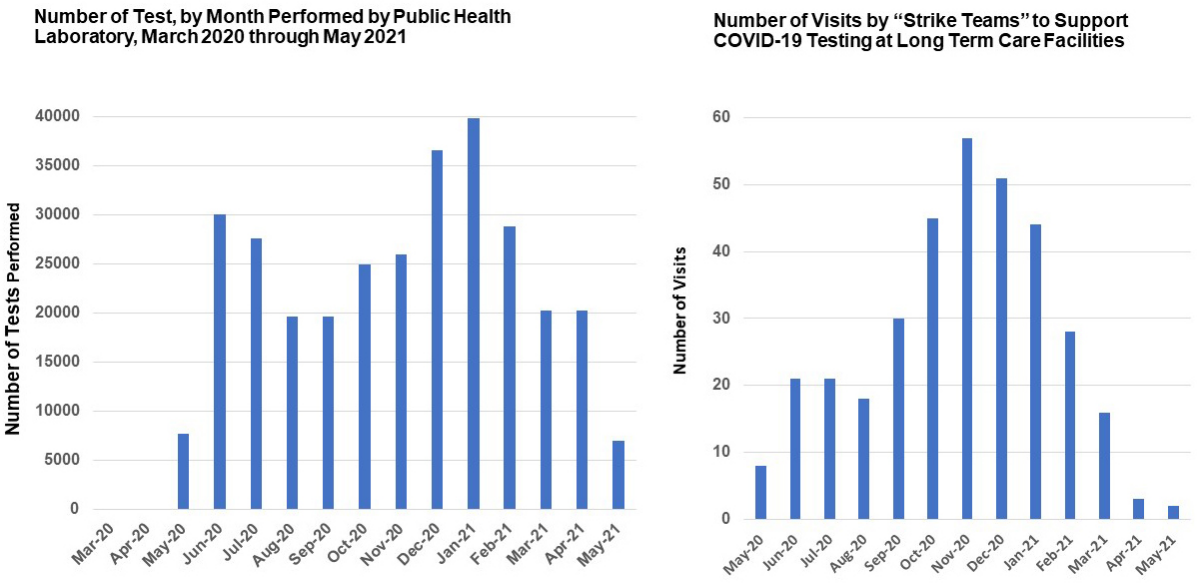

Figure 2. Number of Tests Performed by the Public Health Laboratory and the Number of Visits by “Strike Teams”

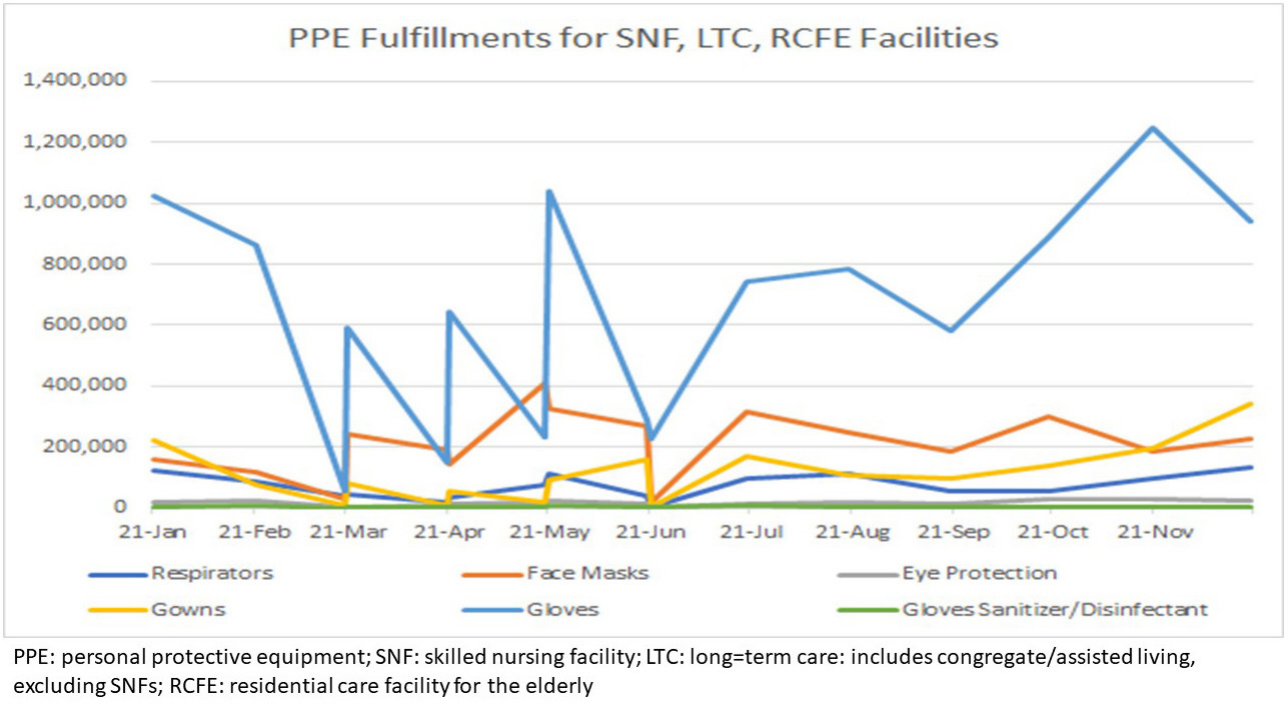

Figure 3. Personal Protective Equipment Fulfillment during COVID-19 Pandemic

**Conclusion:**

The management of the recent COVID-19 outbreaks required a multi-pronged approach. Lessons learned are applicable to other highly transmissible infectious diseases.

**Disclosures:**

**All Authors**: No reported disclosures

